# 2,5-Di­methyl­phenyl quinoline-2-carboxy­l­ate

**DOI:** 10.1107/S160053681400052X

**Published:** 2014-01-15

**Authors:** E. Fazal, Manpreet Kaur, Jerry P. Jasinski, S. Nagarajan, B. S. Sudha

**Affiliations:** a Department of Chemistry, Yuvaraja’s College, Mysore 570 005, India; b Department of Studies in Chemistry, University of Mysore, Manasagangotri, Mysore 570 006, India; c Department of Chemistry, Keene State College, 229 Main Street, Keene, NH 03435-2001, USA; d P.P.S.F.T. Department, Central Food Technplogy Research institute, Mysore 570 005, India

## Abstract

In the title compound, C_18_H_15_NO_2_, the dihedral angle between the mean planes of the quinoline ring system and the phenyl ring is 78.8 (1)°. The mean plane of the carboxyl­ate group is twisted from the mean planes of the quinoline ring system and phenyl ring by 1.5 (9) and 77.6 (4)°, respectively. In the crystal, mol­ecules are linked by weak C—H⋯O inter­actions, generating *C*(8) chains along [001]. Weak π–π stacking inter­actions are also observed [centroid–centroid separation = 3.6238 (12) Å].

## Related literature   

For related structures and background to quinoline derivatives, see: Fazal *et al.* (2014 ▶); Jasinski *et al.* (2010 ▶).
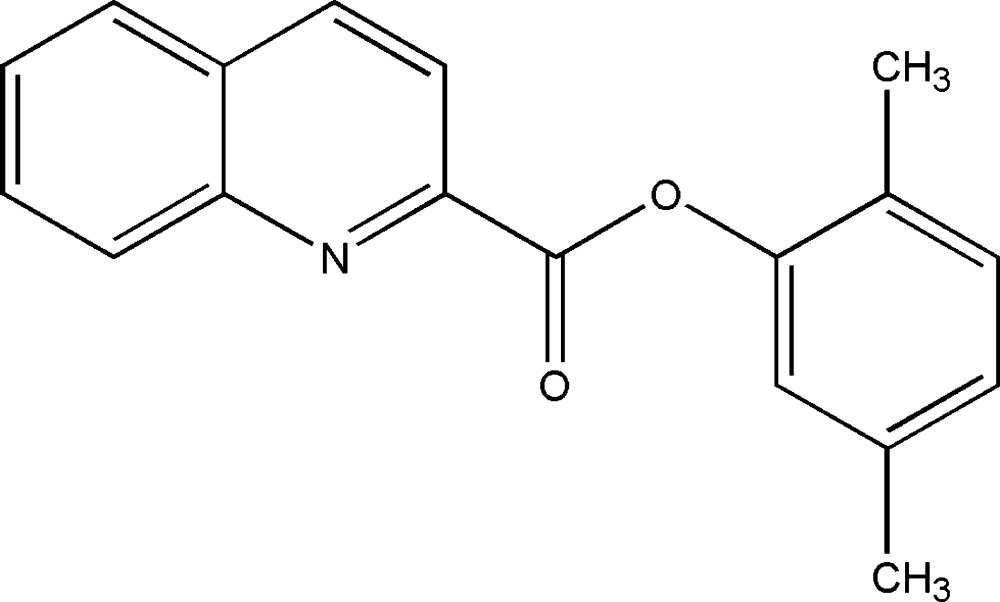


## Experimental   

### 

#### Crystal data  

C_18_H_15_NO_2_
*M* *_r_* = 277.31
Orthorhombic, 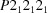

*a* = 8.2261 (3) Å
*b* = 11.6007 (5) Å
*c* = 14.5738 (5) Å
*V* = 1390.76 (9) Å^3^
*Z* = 4
Cu *K*α radiation
μ = 0.69 mm^−1^
*T* = 173 K
0.46 × 0.34 × 0.18 mm

#### Data collection  

Agilent Gemini EOS diffractometer
Absorption correction: multi-scan (*CrysAlis PRO*; Agilent, 2012 ▶) *T*_min_ = 0.711, *T*_max_ = 1.000
8332 measured reflections
2721 independent reflections
2585 reflections with *I* > 2σ(*I*)
*R*_int_ = 0.031

#### Refinement  

*R*[*F* ^2^ > 2σ(*F* ^2^)] = 0.035
*wR*(*F* ^2^) = 0.101
*S* = 1.05
2721 reflections
193 parameters
H-atom parameters constrained
Δρ_max_ = 0.18 e Å^−3^
Δρ_min_ = −0.16 e Å^−3^
Absolute structure: Flack parameter determined using 1049 quotients [(*I* ^+^)−(*I* ^−^)]/[(*I* ^+^)+(*I* ^−^)] (Parsons *et al.*, 2013 ▶)
Absolute structure parameter: −0.04 (16)

### 

Data collection: *CrysAlis PRO* (Agilent, 2012 ▶); cell refinement: *CrysAlis PRO*; data reduction: *CrysAlis RED* (Agilent, 2012 ▶); program(s) used to solve structure: *SUPERFLIP* (Palatinus & Chapuis, 2007 ▶); program(s) used to refine structure: *SHELXL2012* (Sheldrick, 2008 ▶); molecular graphics: *OLEX2* (Dolomanov *et al.*, 2009 ▶); software used to prepare material for publication: *OLEX2*.

## Supplementary Material

Crystal structure: contains datablock(s) I. DOI: 10.1107/S160053681400052X/hb7183sup1.cif

Structure factors: contains datablock(s) I. DOI: 10.1107/S160053681400052X/hb7183Isup2.hkl

Supporting information file. DOI: 10.1107/S160053681400052X/hb7183Isup3.cml

CCDC reference:

Additional supporting information:  crystallographic information; 3D view; checkCIF report

## Figures and Tables

**Table 1:** Hydrogen-bond geometry (Å, °)

*D*—H⋯*A*	*D*—H	H⋯*A*	*D*⋯*A*	*D*—H⋯*A*
C17—H17*B*⋯O1^i^	0.98	2.49	3.389 (3)	152

Symmetry code: (i) 
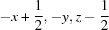
.

**Fig. 1.**
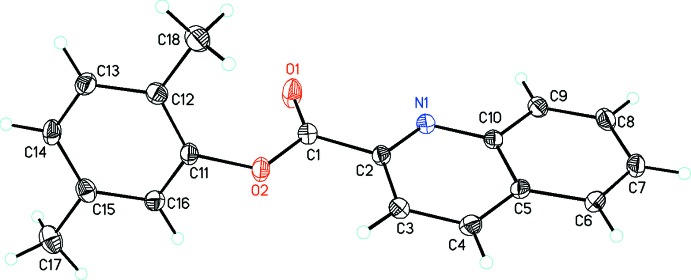
ORTEP drawing of (I) (C18H15NO2) showing 30% probability displacement ellipsoids.

**Fig. 2.**
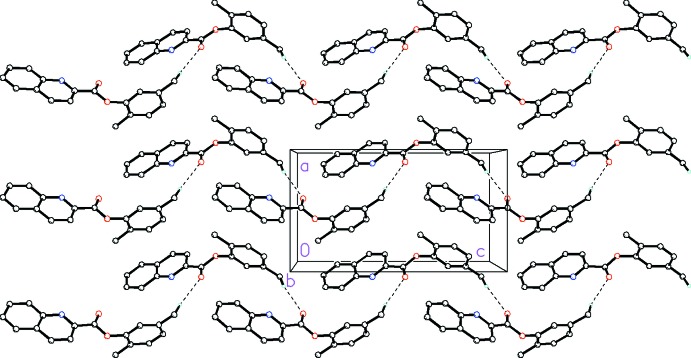
Molecular packing for (I) viewed along the b axis. Dashed lines indicate weak C17—H17B···O1 intermolecular interactions making chains along [0 0 1] and influence the crystal packing. The remaining H atoms have been removed for clarity.

## supplementary crystallographic information

### 1. Comment

Following our recent report on 2-isopropyl-5-methylcyclohexyl quinoline-2-carboxylate (Fazal *et al.*, 2014), we now describe the crystal structure of the title compound, (I), The synthesis, crystal structures and theoretical studies of four Schiff bases derived from 4-hydrazinyl-8-(trifluoromethyl) quinoline (Jasinski *et al.*, 2010) have also been reported.

The dihedral angle between the mean planes of the quinoline ring and the phenyl ring is 78.8 (1)° (Fig. 1). The mean plane of the carboxylate group is twisted from the mean planes of the quinoline ring and phenyl ring by 1.5 (9)° and 77.6 (4)°, respectively. The crystal packing is influenced by weak C17—H17B···O1 interactions making chains along [0 0 1](Fig. 2). In addition, weak Cg2–Cg3 π–π interactions are observed (Cg2–Cg3 = 3.6238 (12)Å; Cg2 = C5–C10; Cg3 = C11–C16; 1/2 + x, 1/2 - y, 1 - z).

### 2. Experimental

To a mixture of 1.73 g (10 mmole) of quinaldic acid and 1.56 g (10 mmole) of 2,5-dimethylphenol in a round-bottomed flask fitted with a reflux condenser with a drying tube was added 0.15 g (10 mmole) of phosphorous oxychloride. The mixture was heated with occasional swirling, and temperature maintained at 348-353 K. At the end of eight hours the reaction mixture was poured in to a solution of 2 g of sodium bicarbonate in 25 ml of water. The precipitated ester was collected on a filter and washed with water. The yield of crude, air dried 2,5-dimethylphenyl quinoline-2-carboxylate was 1.71 to 1.85 g (65-70 %). Irregular colourless chunks were obtained by recrystallization from absolute ethanol solution by slow evaporation.

### 3. Refinement

All of the H atoms were placed in their calculated positions and then refined using the riding model with Atom—H lengths of 0.95Å (CH) or 0.98Å (CH_3_). Isotropic displacement parameters for these atoms were set to 1.2 (CH) or 1.5 (CH_3_) times *U*_eq_ of the parent atom. Idealised Me refined as rotating group.

### Crystal data

**Table:**

C_18_H_15_NO_2_	*D*_x_ = 1.324 Mg m^−^^3^
*M**_r_* = 277.31	Cu *K*α radiation, λ = 1.54184 Å
Orthorhombic, *P*2_1_2_1_2_1_	Cell parameters from 3982 reflections
*a* = 8.2261 (3) Å	θ = 3.0–72.4°
*b* = 11.6007 (5) Å	µ = 0.69 mm^−^^1^
*c* = 14.5738 (5) Å	*T* = 173 K
*V* = 1390.76 (9) Å^3^	Irregular, colourless
*Z* = 4	0.46 × 0.34 × 0.18 mm
*F*(000) = 584	

### Data collection

**Table:**

Agilent Gemini EOS diffractometer	2721 independent reflections
Radiation source: Enhance (Cu) X-ray Source	2585 reflections with *I* > 2σ(*I*)
Detector resolution: 16.0416 pixels mm^-1^	*R*_int_ = 0.031
ω scans	θ_max_ = 72.6°, θ_min_ = 4.9°
Absorption correction: multi-scan (*CrysAlis PRO*; Agilent, 2012)	*h* = −6→10
*T*_min_ = 0.711, *T*_max_ = 1.000	*k* = −14→13
8332 measured reflections	*l* = −17→17

### Refinement

**Table:**

Refinement on *F*^2^	H-atom parameters constrained
Least-squares matrix: full	*w* = 1/[σ^2^(*F*_o_^2^) + (0.0609*P*)^2^ + 0.1748*P*] where *P* = (*F*_o_^2^ + 2*F*_c_^2^)/3
*R*[*F*^2^ > 2σ(*F*^2^)] = 0.035	(Δ/σ)_max_ = 0.008
*wR*(*F*^2^) = 0.101	Δρ_max_ = 0.18 e Å^−^^3^
*S* = 1.05	Δρ_min_ = −0.16 e Å^−^^3^
2721 reflections	Extinction correction: *SHELXL2012* (Sheldrick, 2008), Fc^*^=kFc[1+0.001xFc^2^λ^3^/sin(2θ)]^-1/4^
193 parameters	Extinction coefficient: 0.0027 (7)
0 restraints	Absolute structure: Flack parameter determined using 1049 quotients [(*I*^+^)-(*I*^-^)]/[(*I*^+^)+(*I*^-^)] (Parsons *et al.*, 2013)
Primary atom site location: structure-invariant direct methods	Absolute structure parameter: −0.04 (16)
Hydrogen site location: inferred from neighbouring sites	

### Special details

**Table:**

Geometry. All esds (except the esd in the dihedral angle between two l.s. planes) are estimated using the full covariance matrix. The cell esds are taken into account individually in the estimation of esds in distances, angles and torsion angles; correlations between esds in cell parameters are only used when they are defined by crystal symmetry. An approximate (isotropic) treatment of cell esds is used for estimating esds involving l.s. planes.

### Fractional atomic coordinates and isotropic or equivalent isotropic displacement parameters (Å^2^)

**Table:**

	*x*	*y*	*z*	*U*_iso_*/*U*_eq_	
O1	0.4090 (2)	0.00776 (14)	0.45505 (11)	0.0439 (4)	
O2	0.5803 (2)	0.13777 (12)	0.39481 (9)	0.0330 (4)	
N1	0.3984 (2)	0.13265 (14)	0.61466 (11)	0.0259 (4)	
C1	0.4863 (2)	0.09441 (16)	0.46254 (13)	0.0274 (4)	
C2	0.4902 (2)	0.16990 (15)	0.54669 (12)	0.0248 (4)	
C3	0.5847 (2)	0.27162 (16)	0.54988 (13)	0.0276 (4)	
H3	0.6468	0.2956	0.4984	0.033*	
C4	0.5840 (3)	0.33438 (17)	0.62920 (14)	0.0292 (4)	
H4	0.6464	0.4031	0.6334	0.035*	
C5	0.4907 (3)	0.29728 (16)	0.70485 (13)	0.0265 (4)	
C6	0.4872 (3)	0.35546 (17)	0.79056 (14)	0.0321 (4)	
H6	0.5502	0.4232	0.7992	0.038*	
C7	0.3934 (3)	0.31404 (19)	0.86066 (14)	0.0349 (5)	
H7	0.3927	0.3529	0.9180	0.042*	
C8	0.2979 (3)	0.21456 (19)	0.84884 (14)	0.0334 (5)	
H8	0.2323	0.1877	0.8981	0.040*	
C9	0.2982 (3)	0.15600 (17)	0.76741 (14)	0.0296 (4)	
H9	0.2325	0.0893	0.7601	0.035*	
C10	0.3968 (2)	0.19518 (16)	0.69387 (13)	0.0252 (4)	
C11	0.5828 (2)	0.07494 (17)	0.31151 (12)	0.0271 (4)	
C12	0.6794 (2)	−0.02221 (17)	0.30398 (14)	0.0286 (4)	
C13	0.6803 (3)	−0.07618 (18)	0.21885 (14)	0.0318 (4)	
H13	0.7445	−0.1435	0.2107	0.038*	
C14	0.5903 (3)	−0.03456 (18)	0.14550 (14)	0.0326 (5)	
H14	0.5932	−0.0740	0.0884	0.039*	
C15	0.4960 (2)	0.06388 (18)	0.15446 (13)	0.0313 (4)	
C16	0.4932 (3)	0.11850 (17)	0.23933 (14)	0.0294 (4)	
H16	0.4294	0.1860	0.2477	0.035*	
C17	0.4019 (3)	0.1117 (2)	0.07440 (16)	0.0452 (6)	
H17A	0.3427	0.1809	0.0938	0.068*	
H17B	0.3245	0.0537	0.0525	0.068*	
H17C	0.4772	0.1318	0.0248	0.068*	
C18	0.7792 (3)	−0.0662 (2)	0.38316 (16)	0.0400 (5)	
H18A	0.8297	−0.0011	0.4151	0.060*	
H18B	0.8640	−0.1180	0.3601	0.060*	
H18C	0.7088	−0.1083	0.4258	0.060*	

### Atomic displacement parameters (Å^2^)

**Table:**

	*U*^11^	*U*^22^	*U*^33^	*U*^12^	*U*^13^	*U*^23^
O1	0.0543 (10)	0.0445 (9)	0.0330 (8)	−0.0212 (8)	0.0128 (8)	−0.0122 (7)
O2	0.0444 (8)	0.0311 (7)	0.0235 (7)	−0.0073 (6)	0.0076 (6)	−0.0061 (5)
N1	0.0281 (8)	0.0265 (8)	0.0232 (8)	0.0012 (7)	0.0003 (7)	−0.0005 (6)
C1	0.0283 (9)	0.0294 (9)	0.0246 (9)	−0.0001 (8)	0.0006 (8)	−0.0011 (7)
C2	0.0270 (8)	0.0262 (9)	0.0211 (8)	0.0032 (8)	−0.0015 (8)	−0.0008 (7)
C3	0.0305 (9)	0.0273 (9)	0.0251 (9)	0.0009 (8)	0.0008 (8)	0.0019 (7)
C4	0.0327 (9)	0.0237 (9)	0.0311 (10)	−0.0010 (8)	−0.0015 (8)	−0.0002 (8)
C5	0.0308 (9)	0.0238 (9)	0.0248 (9)	0.0061 (8)	−0.0032 (8)	−0.0015 (7)
C6	0.0415 (11)	0.0251 (9)	0.0296 (10)	0.0033 (9)	−0.0039 (9)	−0.0041 (7)
C7	0.0453 (11)	0.0359 (11)	0.0234 (9)	0.0113 (10)	−0.0012 (9)	−0.0056 (8)
C8	0.0372 (10)	0.0397 (11)	0.0232 (9)	0.0093 (9)	0.0029 (9)	0.0028 (8)
C9	0.0302 (9)	0.0321 (10)	0.0264 (10)	0.0025 (8)	−0.0001 (8)	0.0021 (8)
C10	0.0269 (9)	0.0255 (9)	0.0234 (8)	0.0052 (8)	−0.0015 (7)	0.0004 (7)
C11	0.0336 (10)	0.0254 (9)	0.0222 (8)	−0.0067 (8)	0.0048 (8)	−0.0033 (7)
C12	0.0289 (9)	0.0281 (9)	0.0288 (10)	−0.0035 (8)	0.0038 (8)	0.0017 (8)
C13	0.0338 (10)	0.0272 (9)	0.0344 (10)	−0.0004 (9)	0.0087 (9)	−0.0026 (8)
C14	0.0377 (10)	0.0345 (11)	0.0256 (9)	−0.0093 (9)	0.0046 (9)	−0.0091 (8)
C15	0.0300 (9)	0.0375 (10)	0.0263 (9)	−0.0080 (9)	0.0015 (9)	0.0012 (8)
C16	0.0316 (9)	0.0271 (9)	0.0296 (10)	0.0012 (9)	0.0056 (8)	0.0005 (7)
C17	0.0443 (12)	0.0608 (15)	0.0306 (11)	−0.0007 (12)	−0.0047 (10)	0.0035 (10)
C18	0.0411 (12)	0.0450 (12)	0.0338 (11)	0.0028 (10)	0.0004 (10)	0.0049 (10)

### Geometric parameters (Å, º)

**Table:**

O1—C1	1.194 (2)	C9—H9	0.9500
O2—C1	1.351 (2)	C9—C10	1.419 (3)
O2—C11	1.416 (2)	C11—C12	1.383 (3)
N1—C2	1.319 (2)	C11—C16	1.380 (3)
N1—C10	1.363 (2)	C12—C13	1.390 (3)
C1—C2	1.507 (2)	C12—C18	1.505 (3)
C2—C3	1.414 (3)	C13—H13	0.9500
C3—H3	0.9500	C13—C14	1.387 (3)
C3—C4	1.366 (3)	C14—H14	0.9500
C4—H4	0.9500	C14—C15	1.387 (3)
C4—C5	1.411 (3)	C15—C16	1.390 (3)
C5—C6	1.420 (2)	C15—C17	1.507 (3)
C5—C10	1.423 (3)	C16—H16	0.9500
C6—H6	0.9500	C17—H17A	0.9800
C6—C7	1.368 (3)	C17—H17B	0.9800
C7—H7	0.9500	C17—H17C	0.9800
C7—C8	1.407 (3)	C18—H18A	0.9800
C8—H8	0.9500	C18—H18B	0.9800
C8—C9	1.367 (3)	C18—H18C	0.9800
			
C1—O2—C11	116.31 (15)	C9—C10—C5	119.47 (17)
C2—N1—C10	117.84 (16)	C12—C11—O2	119.72 (17)
O1—C1—O2	123.44 (18)	C16—C11—O2	117.19 (17)
O1—C1—C2	125.08 (18)	C16—C11—C12	123.00 (17)
O2—C1—C2	111.47 (16)	C11—C12—C13	116.18 (18)
N1—C2—C1	114.13 (16)	C11—C12—C18	121.93 (18)
N1—C2—C3	124.32 (17)	C13—C12—C18	121.89 (19)
C3—C2—C1	121.55 (17)	C12—C13—H13	119.1
C2—C3—H3	121.0	C14—C13—C12	121.88 (19)
C4—C3—C2	118.05 (18)	C14—C13—H13	119.1
C4—C3—H3	121.0	C13—C14—H14	119.6
C3—C4—H4	120.0	C15—C14—C13	120.84 (18)
C3—C4—C5	120.07 (18)	C15—C14—H14	119.6
C5—C4—H4	120.0	C14—C15—C16	117.95 (19)
C4—C5—C6	123.59 (18)	C14—C15—C17	121.20 (19)
C4—C5—C10	117.47 (16)	C16—C15—C17	120.8 (2)
C6—C5—C10	118.93 (17)	C11—C16—C15	120.15 (19)
C5—C6—H6	120.0	C11—C16—H16	119.9
C7—C6—C5	120.08 (19)	C15—C16—H16	119.9
C7—C6—H6	120.0	C15—C17—H17A	109.5
C6—C7—H7	119.6	C15—C17—H17B	109.5
C6—C7—C8	120.80 (18)	C15—C17—H17C	109.5
C8—C7—H7	119.6	H17A—C17—H17B	109.5
C7—C8—H8	119.6	H17A—C17—H17C	109.5
C9—C8—C7	120.9 (2)	H17B—C17—H17C	109.5
C9—C8—H8	119.6	C12—C18—H18A	109.5
C8—C9—H9	120.1	C12—C18—H18B	109.5
C8—C9—C10	119.82 (19)	C12—C18—H18C	109.5
C10—C9—H9	120.1	H18A—C18—H18B	109.5
N1—C10—C5	122.21 (17)	H18A—C18—H18C	109.5
N1—C10—C9	118.32 (17)	H18B—C18—H18C	109.5
			
O1—C1—C2—N1	−0.1 (3)	C6—C5—C10—C9	2.1 (3)
O1—C1—C2—C3	−179.8 (2)	C6—C7—C8—C9	0.9 (3)
O2—C1—C2—N1	178.98 (16)	C7—C8—C9—C10	0.5 (3)
O2—C1—C2—C3	−0.7 (3)	C8—C9—C10—N1	177.87 (18)
O2—C11—C12—C13	−177.39 (17)	C8—C9—C10—C5	−1.9 (3)
O2—C11—C12—C18	2.0 (3)	C10—N1—C2—C1	179.07 (16)
O2—C11—C16—C15	177.19 (17)	C10—N1—C2—C3	−1.3 (3)
N1—C2—C3—C4	1.5 (3)	C10—C5—C6—C7	−0.8 (3)
C1—O2—C11—C12	−79.9 (2)	C11—O2—C1—O1	0.5 (3)
C1—O2—C11—C16	103.4 (2)	C11—O2—C1—C2	−178.63 (15)
C1—C2—C3—C4	−178.88 (17)	C11—C12—C13—C14	0.3 (3)
C2—N1—C10—C5	−0.3 (3)	C12—C11—C16—C15	0.6 (3)
C2—N1—C10—C9	179.87 (17)	C12—C13—C14—C15	0.5 (3)
C2—C3—C4—C5	−0.1 (3)	C13—C14—C15—C16	−0.8 (3)
C3—C4—C5—C6	177.96 (18)	C13—C14—C15—C17	178.1 (2)
C3—C4—C5—C10	−1.4 (3)	C14—C15—C16—C11	0.2 (3)
C4—C5—C6—C7	179.9 (2)	C16—C11—C12—C13	−0.9 (3)
C4—C5—C10—N1	1.6 (3)	C16—C11—C12—C18	178.5 (2)
C4—C5—C10—C9	−178.58 (18)	C17—C15—C16—C11	−178.65 (19)
C5—C6—C7—C8	−0.7 (3)	C18—C12—C13—C14	−179.00 (19)
C6—C5—C10—N1	−177.74 (18)		

### Hydrogen-bond geometry (Å, º)

**Table:**

*D*—H···*A*	*D*—H	H···*A*	*D*···*A*	*D*—H···*A*
C17—H17*B*···O1^i^	0.98	2.49	3.389 (3)	152

Symmetry code: (i) −*x*+1/2, −*y*, *z*−1/2.
